# GIPR Signaling in Immune Cells Maintains Metabolically Beneficial Type 2 Immune Responses in the White Fat From Obese Mice

**DOI:** 10.3389/fimmu.2021.643144

**Published:** 2021-02-25

**Authors:** Irina Efimova, Inbar Steinberg, Isabel Zvibel, Anat Neumann, Dana Fernanda Mantelmacher, Daniel J. Drucker, Sigal Fishman, Chen Varol

**Affiliations:** ^1^ The Research Center for Digestive Tract and Liver Diseases, Tel-Aviv Sourasky Medical Center Affiliated to Tel-Aviv University, Tel Aviv, Israel; ^2^ Department of Clinical Microbiology and Immunology, Sackler School of Medicine, Tel-Aviv University, Tel-Aviv, Israel; ^3^ The Lunenfeld-Tanenbaum Research Institute, Mount Sinai Hospital, University of Toronto, Toronto, ON, Canada

**Keywords:** glucose-dependent insulinotropic polypeptide receptor, obesity, type 2 immunity, white adipose tissue, S100A8/A9

## Abstract

Glucose-dependent insulinotropic polypeptide (GIP) communicates information on energy availability from the gut to peripheral tissues. Disruption of its signaling in myeloid immune cells during high-fat diet (HFD)-induced obesity impairs energy homeostasis due to the unrestrained metabolically deleterious actions of S100A8/A9 alarmin. White adipose tissue (WAT) type 2 immune cell networks are important for maintaining metabolic and energy homeostasis and limiting obesity-induced inflammation. Nevertheless, the consequences of losing immune cell GIP receptor (GIPR) signaling on type 2 immunity in WAT remains unknown. Bone marrow (BM) chimerism was used to generate mice with GIPR (*Gipr^-/-^* BM) and GIPR*/*S100A8/A9 *(Gipr^-/-^*/*S100a9^-/-^* BM) deletion in immune cells. These mice were subjected to short (5 weeks) and progressive (14 weeks) HFD regimens. GIPR-deficiency was also targeted to myeloid cells by crossing *Gipr^fl/fl^* mice and *Lyz2^cre/+^* mice (*LysM^ΔGipr^*). Under both short and progressive HFD regimens, *Gipr^-/-^* BM mice exhibited altered expression of key type 2 immune cytokines in the epididymal visceral WAT (epiWAT), but not in subcutaneous inguinal WAT. This was further linked to declined representation of type 2 immune cells in epiWAT, such as group 2 innate lymphoid cells (ILC2), eosinophils, and FOXP3^+^ regulatory T cells (Tregs). Co-deletion of S100A8/A9 in *Gipr^-/-^* immune cells reversed the impairment of type 2 cytokine expression in epiWAT, suggesting a mechanistic role for this alarmin in type 2 immune suppression. *LysM^ΔGipr^* mice on HFD also displayed altered expression of type 2 immune mediators, highlighting that GIPR-deficiency in myeloid immune cells is responsible for the impairment of type 2 immune networks. Finally, abrogated GIPR signaling in immune cells also affected adipocyte fraction cells, inducing their increased production of the beiging interfering cytokine IL-10 and stress- related type 2 cytokine IL-13. Collectively, these findings attribute an important role for GIPR in myeloid immune cells in supporting WAT type 2 immunity.

## Introduction

Integrated immunometabolic responses couple dietary intake, energy utilization, and storage to immune regulation of tissue function, and are therefore essential for the maintenance and restoration of homeostasis (1). Enteroendocrine cells (EECs) are strategically positioned along the entire gastrointestinal tract, where upon sensing ingested food, they produce hormonal messengers that transmit signals of nutrient availability to the brain and to metabolically important peripheral sites to allow the host to prioritize storage and/or utilize substrates for tissue growth, maintenance, and immune responses (2). Two gut hormones, Glucagon-like Peptide-1 (GLP-1) and Glucose-Dependent Insulinotropic Polypeptide (GIP), secreted by gut L and K cells, respectively, have attracted great interest in view of their metabolic roles and therapeutic potential (3, 4). Both hormones are known for their glucose-dependent insulinotropic actions on the pancreas *via* G-protein coupled receptors expressed on pancreatic islet cells (the incretin effect) (3). In addition, they both perform extra-pancreatic actions and differentially control metabolic homeostasis by modulation of satiety and food intake, potentially affecting energy expenditure (5).

Multiple immune cell subsets express receptors for both GLP-1 and GIP, pinpointing the immune system as another target for incretin action (6, 7). However, there is comparatively little comprehension of the precise roles of incretins in the control of immunometabolism. Specifically, the directional importance of GIP in obesity-associated inflammatory responses is surrounded by controversy, with studies reporting both pro- (8, 9) and anti-inflammatory (10, 11) effects in the white adipose tissue (WAT). These opposing results likely reflect the integrated pleiotropic functions of GIP in different tissues and cells, and the utilization in many studies of GIP analogues that can serve as both agonists and antagonists (12). Therefore, studying GIP biology in a cell-specific manner can benefit our comprehension of its immunometabolic pathways. In this respect, we have recently discovered a direct role for GIP in myeloid immune cells critical for controlling inflammation and body weight (11, 13). In contrast to the overall metabolic protection of mice with stimuli global germline inactivation of the GIPR (14), bone marrow (BM) chimeric mice with immune cell-targeted GIPR-deficiency (*Gipr^-/-^* BM) display greater weight gain during HFD feeding, despite similar food intake, concomitantly with increased insulin resistance and hepatic steatosis (13).

Thermogenesis is a process that dissipates energy as heat, and takes place in brown adipose tissue (BAT) and also in brown-like adipocytes (termed “beige” cells) in the inguinal WAT (ingWAT). Beige cells are generated from precursors or mature adipocytes in response to certain stimuli like chronic cold exposure, prolonged β-adrenergic stimulation or increased calorie intake (15, 16). Non-shivering thermogenesis generated by brown adipocytes and beiging is recognized as a relevant human physiological mechanism to offset obesity. The increased weight gain displayed by HFD-fed *Gipr^-/-^* BM is a result of impaired energy expenditure due to reduced ingWAT beiging (13). Targeting GIPR-deficiency to myeloid immune cells further pinpointed a mechanistic role for GIP in restraining the production of the alarmin S100A8/A9 (calprotectin), which hampers adaptive thermogenesis and ingWAT beiging. Nevertheless, the precise comprehension of how disruption of GIPR signaling in immune cell impairs energy balance remains elusive.

Type 2 immune cell networks are important for maintaining metabolic homeostasis and energy balance (17). Accordingly, eosinophil-derived IL-4 was suggested to maintain alternative M2 activation of adipose tissue macrophages (ATMs) to facilitate ingWAT beiging (18, 19), though other studies have challenged the contribution of ATMs to adaptive thermogenesis (20). Group 2 innate lymphoid cells (ILC2s) residing in the lean WAT activate eosinophils *via* IL-5 secretion and polarize ATMs towards M2 phenotype *via* IL-13 production (21, 22). ILC2s have also been shown to directly promote adipocyte beiging *via* production of Methionine-Enkephalin (MetEnk) peptides that upregulate UCP1 expression in adipocytes. The alarmin IL-33 is critical for priming the pro-beiging activity of ILC2s in WAT (21, 23). This type 2 cytokine also promotes proliferation of ST2 (IL-33 receptor)-expressing Tregs (24, 25), and acts directly on adipocytes to promote beiging and thermogenesis, thereby offsetting obesity (24, 26). IL-33 is locally produced within the epididymal visceral adipose tissue (epiWAT) mainly by mesenchymal stromal cell subtypes (27, 28). In light of this imperative crosstalk between metabolism and type 2 immune cells within fat depots, we examined here whether deletion of GIPR signaling in immune cells, and the resulted impairment in energy homeostasis during obesity, is due to the alteration of fat depot type 2 immunity. Indeed, we show here that targeted immune cell GIPR-deficiency results in a significant reduction of type 2 immune cells and pro-beiging mediators in the obese epiWAT, mediated in part through the unrestrained activity of the pro-inflammatory alarmin S100A8/A9. These results highlight a gut-immune axis governed by GIP, coupling nutrient availability to the control of metabolically beneficial type 2 immune responses in the obese epiWAT.

## Materials and Methods

### Animals

C57BL/6J OlaHsd male mice (Envigo, Jerusalem, Israel), *Gipr^-/-^* mice (C57Bl/6 background, provided by Dr Y. Yamada, Akita University, Japan), *S100a9^-/-^* mice (C57Bl/6 background, provided by Prof. T. Vogl, Munster University, Germany), B6.129P2-*Lyz2^tm1(cre)Ifo^* (C57Bl/6 background, Jackson laboratory) and *Gipr^fl/fl^* mice (C57Bl/6 background, provided by Prof. D J Drucker, Mt. Sinai Hospital, University of Toronto, Canada) were maintained in specific pathogen-free animal facility and experiments were performed according to protocols approved by the Animal Care Use Committee of the Sourasky Medical Center. All mice were housed with 12 h light cycles and a constant temperature of 22**°**C, with free access to standard rodent chow (Ctrl, 5% kcal fat, 5010, Lab Diets) and water. For the generation of BM chimeric mice, 6-week-old recipient C57BL/6J OlaHsd male mice were lethally irradiated with 950 rad using a TrueBeam linear accelerator (Varian Medical Systems). The next day, femurs were dissected from 6-weeks-old donor male mice (WT, *Gipr^-/-^* and *Gipr^-/-^/S100a9^-/-^* mice), all born and raised in the same facility. About 5x10^6^ of respective BM cells were injected into the tail vein of the recipients, and mice were allowed to reconstitute their BM-derived immune cells for 6 weeks. After reconstitution, weight-matched mice were fed with HFD (60% kcal from fat, Research Diets, D12492) for the indicated period. Male mice were used to exclude the effect of female hormones and hormonal cycling on insulin secretion and metabolism.

### Cell Isolation of Stromal Vascular and Mature Adipocyte Fractions

Epididymal fat pads (epiWAT) and inguinal subcutaneous fat (ingWAT) were surgically removed from mice, weighted, cut into small pieces and subsequently incubated in a 50 ml conical vial with digestion buffer [DMEM, 12.5 mM HEPES pH 7.4, 2% BSA and 10 mg collagenase type II] for 40 min at 37^0^C in a shaking bath at 150 rpm. The digested tissues were filtered through a 100 μM metal mesh and centrifuged at 1,400 g for 5 min: the lipid-containing mature adipocytes floated to the top, and the stromal vascular fraction (SVF) pelleted at the bottom. Mature adipocyte fraction was collected and stored at -80°C. The SVF pellet was resuspended in red blood cell lysis solution, and recovered cells were washed with cold 2% fetal bovine serum in phosphate-buffered saline (PBS). The SVF pellet was used for flow cytometry analysis and RNA extraction. For RNA isolation, cells were suspended in 1 ml of TRI Reagent (Sigma-Aldrich) and then stored at 80°C until further analysis.

### Flow Cytometry Analysis and Sorting of Adipose Tissue Macrophages

Flow cytometry analysis was performed using BD FACSCanto™ II Cell Analyzer (BD Biosciences), and ATMs were sort-purified using BD FACSAria™ Fusion Cell Sorter (BD Biosciences). After blocking the Fc receptors with CD16/CD32 antibody (Bio-Legend), cell suspensions of WAT were incubated on ice with fluorochrome-conjugated antibodies in FACS buffer. The following antibodies were used to stain cells from mouse tissues: CD45 (30-F11), CD25 (PC61), CD90.2 (30-H12), IL-33Rα/ST2 (DIH9), CD5 (53-7.3), CD19 (1D3/CD19), CD11b (M1/70), CD11c (N418), FcϵRI (MAR-1), NK1.1 (PK136), CD3ϵ (145-2C11), CD49b (DX5), SiglecF (E50-2440), CD31 (390), F4/80 (BM8), CD4 (RM4-5), CD8 (53-6.7), CD25 (PC61). Foxp3 (FJK-16S) was stained using the Foxp3 transcription factor staining buffer set (eBioscience). All antibodies used in flow cytometry were purchased from eBioscience, BioLegend, or BD Biosciences if not indicated otherwise above. Data were analyzed with FlowJo v10.4.1 software (TreeStar).

### Intracellular Cytokine Analysis

To examine IL-10 production in immune cells, we cultured single-cell suspensions of SVF for 4 h ex vivo with phorbol 12-myristate 13-acetate (100 ng/ml; Sigma-Aldrich) and ionomycin (1 ug/ml; Sigma-Aldrich) in the presence of brefeldin A (10 μg/ml; Sigma-Aldrich) in a 37°C incubator (5% CO_2_). Cells were washed with cold PBS and surface-stained before fixation and permeabilization using the Cytofix/Cytoperm Fixation/Permeabilization solution and Perm/Wash buffer according to the manufacturer’s protocol (eBioscience). Intracellular staining was performed with IL-10 (JeS5-16E3) antibody (BioLegend).

### Quantitative Real-Time Polymerase Chain Reaction

Frozen adipose tissue and mature adipocyte fraction were suspended in Qiazol (Qiagen) and homogenized using a Polytron (Kinematica). RNA was isolated using an RNeasy kit (Qiagen) following the manufacturer`s instructions. The SVF cells and sorted cells were homogenized in TRI Reagent (Sigma-Aldrich) and RNA-extracted according to the manufacturer`s protocol. Total RNA was reverse-transcribed using the High capacity cDNA RT kit (Applied Biosystems). Real time RT-PCR was performed with the Fast SYBR Green Master Mix (Applied Biosystems) using the Corbett rotor light cycler. Quantification of the PCR signals of each sample was performed by the ΔCt method and normalized to the housekeeping gene *Rplp0*. The following were the primer sets used: *Il33* F, 5’-ATTTCCCCGGCAAAGTTC-3’; *Il33* R, 5’- CTTATGGTGAGGCCAGAACG-3’; *Penk* F, 5’- GGAGGCTTCATGAAACGGTA -3’; *Penk* R, 5’-TTCAGCAGATCGGAGGAGTT-3’; *Il5* F, 5’- CACCAGCTATGCATTGGAG A-3’; *Il5* R, 5’- TAATCCAGGAACTGCCTCGT-3’; *Il13* F, 5’-TCCAATTGCAATGCCATCT A-3’; *Il13* R, 5’- TGGGCTACTTCGATTTTGGT-3’; *Il10* F, 5’- TGCTATGCTGCCTGCTCT TA-3’; *Il10* R, 5’-ATGTTGTCCAGCTGGTCCTT-3’; *Atf4* F, 5’- CCTTCGACCAGTCGGGT TTG-3’; *Atf4* R, 5’- CTGTCCCGGAAAAGGCAT-3’; *Ddit3* F, 5’-CTGGAAGCCTGGTATG AGGAT-3’; *Ddit3* R, 5’- CAGGGTCAAGAGTAGTGAAGGT -3’; *Xbp1s* F, 5’- GAGTCCGC AGCAGGTG-3’, *Xbp1s* R, 5’- GTGTCAGAGTCCATGGGA -3’; *Rplp0* F, 5’- CAACCCAGC TCTGGAGAAAC-3’; *Rplp0* R, 5’- GTTCTGAGCTGGCACAGTGA -3’.

### Protein Immunoblots

Total protein from epiWAT tissue and epiWAT adipocyte fraction was extracted by homogenization in ice-cold RIPA buffer (PBS, 1% Igepal, 0.5% sodium deoxycholate, 0.1% sodium dodecyl sulfate, protease and phosphatase inhibitors cocktails 1:100). Homogenates were centrifuged for 20 min at 14,000 g, supernatants were collected and extracts were normalized to total protein content. Proteins were separated by sodium dodecyl sulfate polyacrylamide gel electrophoresis (SDS-PAGE), blotted onto nitrocellulose and blots were blocked for 1 h in 5% milk. Blots were incubated overnight at 4°C with antibodies to pEIF2α (Cell Signaling #119A11), XBP1s (BioLegend #647501), pIRE (Cell Signaling #3294) or P97 (BioLegend #636801), and then incubated with horseradish peroxidase-conjugated secondary antibodies and subjected to chemi-luminescent detection using the Micro Chemiluminescent imaging system (DNR Bio Imaging Systems). Densitometry was performed using the Image J software and expression of the proteins normalized to the expression of housekeeping protein P97.

### dAla2-GIP Administration

C57BL/6JOlaHsd 6 weeks old weight matched male mice were group-housed on 12:12 h light-dark cycle at 22°C, with free access to food and water. Mice were maintained on the HFD for 14 weeks and received a single daily i.p. injection of human dAla2-GIP analogue (60 nmol/kg body weight) (synthesized by Bio-Synthesis, Lewisville, Tx) or vehicle (saline) during the last 8 weeks of the obesogenic diet. Separate groups of mice were maintained on regular chow (RC) and received daily i.p. injections of human dAla2-GIP (60 nmol/kg body weight) or vehicle (saline) for 6 consecutive days. All injections were performed in the light cycle.

### Cold Exposure

For cold-challenge experiments, individually housed mice with free access to chow were exposed to 4^0^C in a temperature-controlled refrigerator (4–8°C) for 24 h. Rectal temperature was measured at indicated times (0 and 5 h) using a rectal thermometer (Vega Technologies, Taiwan). After completion of the challenge, mice were sacrificed, and their WAT samples were used for flow cytometry analysis or dissected and stored at -80°C.

### Statistical Analysis

Statistical significance was determined between two groups using an unpaired two-tailed Student’s t-test using GraphPad Prism 6.0 and differences between more than two groups were examined by one-way ANOVA with a Tukey post-hoc test. Significance was defined if p-value was less than 0.05 as following: * p < 0.05; ** p < 0.01; *** p < 0.001. Date are routinely represented as mean ± SEM, and the number of replicates per experiment can be found in the figure legends.

## Results

### GIPR-Deficiency in Immune Cells Alters Expression of type 2 Immune Mediators in the Obese White Adipose Tissue

**Figure 1:**
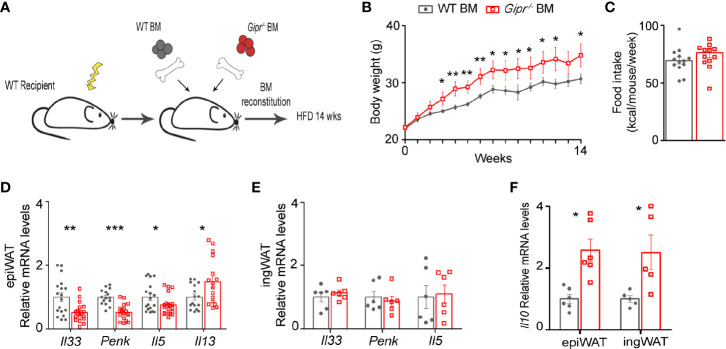
Impaired production of type 2 immune mediators in the obese epiWAT of *Gipr^-/-^* BM mice. **(A)** Scheme illustrating experimental design. Lethally irradiated WT mice were reconstituted by transplantation of WT BM (gray circles) or *Gipr^-/-^* BM (red squares) for 6 weeks and then fed a HFD for 14 weeks. **(B)** Weekly measurements of body weight (g) (n≥6). **(C)** Average weekly food consumption (kcal) per mouse during a period of 14 weeks. **(D–E)** Expression of key type 2 immune mediators in **(D)** epiWAT (n ≥17) and **(E)** ingWAT (n=6) after 14 weeks of HFD, as analyzed by qRT-PCR and normalized to *Rplp0*. **(F)** Relative expression of *Il10* in epiWAT and ingWAT as determined by qRT-PCR. Data is compiled from three individual experiments **(D)** or from single individual experiment **(B, C, E, F),** and presented as mean ± SEM with significance: *p ≤ 0.05, **p ≤ 0.01, and ***p ≤ 0.001, unpaired two-tailed *t*-test, comparing between WT BM and *Gipr^-/-^* BM groups. WT, wild type mice; BM, bone marrow; HFD, high-fat diet; epiWAT, epididymal WAT; ingWAT, subcutaneous inguinal WAT.

WAT is densely populated by various type 2 immune cells that maintain metabolic homeostasis and energy balance, but become dysregulated in obesity (17). Mice with targeted GIPR-deficiency to immune cells exhibit a deteriorated metabolic profile and significant myelopoiesis, concomitantly with impaired energy expenditure and ingWAT beiging under the setting of HFD-induced obesity (13). Hence, we hypothesized that their impaired beiging response may be due to the alteration in metabolically beneficial type 2 immune circuits. To explore this possibility, we implemented a BM chimerism approach to target GIPR-deficiency to immune cells. The BM of lethally irradiated wild type (WT) recipient mice was differentially reconstituted by transplantation of WT (WT BM*)* or *Gipr^-/-^* BM (*Gipr^-/-^* BM). Following immune cell reconstitution (6 weeks), weight-matched groups were subjected to 14 weeks of HFD (**Figure 1A**). In agreement with our previous findings (13), *Gipr^-/-^* BM mice displayed significantly increased weight gain starting already at the 3rd week of HFD feeding (**Figure 1B** **)**, without a change in food consumption (**Figure 1C**). Further analysis revealed dysregulated gene expression of key type 2 immune mediators in the epiWAT of *Gipr^-/-^* BM mice. Notable was the impaired expression of IL-33 (*Il33*) and MetEnk (*Penk*) in epiWAT (**Figure 1D**), key pro-beiging mediators acting upstream on both adipocytes and local type 2 immune cells (21, 23–26). There was also a reduction of IL-5 mRNA (*Il5*) (**Figure 1D**), which was previously linked with the pro-beiging activity of ILC2 (23). In contrast, the expression of another type 2 immune mediator, IL-13, was upregulated in epiWAT (**Figure 1D**). These alterations were most prominent in epiWAT and were not observed in ingWAT (**Figure 1E**).

IL-10 is a type 2 cytokine classically known for its immunosuppressive role in immunity. In WAT, it was suggested as an essential promotor of M2-alternatively activated phenotype of ATMs (29–32). Yet, emerging evidence points to IL-10 as a pivotal suppressor of adipocyte thermogenesis capable of directly repressing the transcription of thermogenic genes in adipocytes (33). In this respect, IL-10 gene expression (*Il10*) was increased in the *Gipr^-/-^* BM epiWAT, and also in the ingWAT at 14 weeks of HFD (**Figure 1F**). Overall, these results suggest a role for GIPR signaling in immune cells as a promoter of type 2 immunity in the epiWAT.

### GIPR-Deficiency in Immune Cells Reduces the Representation of Type 2 Immune Cells in the Obese White Adipose Tissue

**Figure 2:**
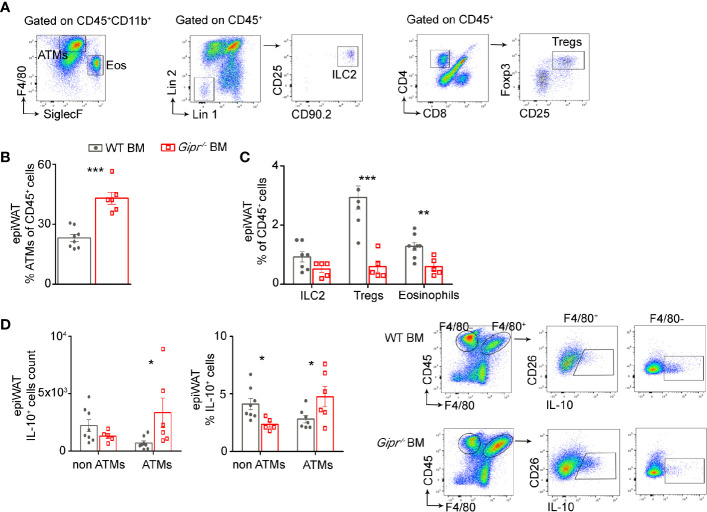
Reduced frequency of type 2 immune cells in the obese epiWAT of *Gipr^-/-^* BM mice. Mice were reconstituted with WT BM (gray circles) or *Gipr^-/-^* BM (red squares) for 6 weeks and then fed a HFD for 14 weeks (n≥6). **(A)** Representative flow cytometry dot plots showing gating strategy for immune cell populations first gated on living CD45^+^ cells from epiWAT. ATMs were identified as CD11b^+^F4/80^+^CD64^+^. Eosinophils were defined as CD11b^+^SiglecF^+^SSC^hi^; ILC2 as Lin^-^CD25^+^CD90.2^+^IL-33R^+^ and Tregs as CD4^+^CD8^-^CD25^+^FOXP3^+^. **(B)** Graph showing flow cytometry assessment of ATMs fraction out of total CD45^+^. **(C)** Fraction of ILC2, Tregs and Eos out of total CD45^+^ cell population. **(D)** Proportion and numbers of IL-10^+^ cells among ATMs (F4/80^+^) and non-ATMs (F4/80^-^) cell populations, normalized to tissue mass in the epiWAT of HFD-fed chimeras. Right panel, representative flow cytometry images depicting the IL-10^+^F4/80^+^ and IL-10^+^F4/80^-^ immune cells. Data are analyzed by unpaired, two-tailed *t*-test, comparing between WT BM and *Gipr^-/-^* BM groups, and are presented as mean ± SEM. *p ≤ 0.05, **p ≤ 0.01 and ***p < 0.001 ATMs, adipose tissue macrophages; ILC2, innate lymphoid cells type 2; Eos, eosinophils; Tregs, T-regulatory cells.

Given the impairment in the production of type 2 immune mediators in the epiWAT during HFD induced by GIPR-deficiency in immune cells (**Figure 1**), we next examined the representation of key associated type 2 immune cells in the epiWAT stromal vascular fracture (SVF) of WT BM vs. *Gipr^-/-^* BM mice. Gating strategy for the various immune populations is shown in **Figure 2A**. In agreement with our earlier studies (13), the fraction (%) of ATMs (defined as CD11b^+^F4/80^+^CD64^+^ cells) out of living CD45^+^ was increased by about 2-fold in the *Gipr^-/-^* BM epiWAT (**Figure 2B**). In contrast, the fraction of eosinophils (defined as CD11b^+^SiglecF^+^SSC^hi^) was reduced by two-fold (**Figure 2C**). Previous studies indicated an important role for IL-33 in maintaining WAT ILC2 and Tregs (21, 23–25). In alignment with the reduction in IL-33 in the epiWAT of *Gipr^-/-^* BM mice at 14 weeks of HFD (**Figure 1D**), the representation of ILC2 (defined as Lin^-^CD25^+^CD90.2^+^IL-33R^+^) trended lower (**Figure 2C**), and there was a five-fold decline in the levels of Tregs (defined as CD4^+^CD8^-^CD25^+^FOXP3^+^) (**Figure 2C**). IL-10 is produced by multiple immune cells in the WAT SVF, including ATMs (34), dendritic cells (35), Tregs (36), and regulatory B cells (37). Intracellular staining revealed increased expression of IL-10 in F4/80^+^ ATMs in the epiWAT of *Gipr^-/-^* BM mice, while it was reduced among F4/80^-^ immune cells (**Figure 2D**). Hence, the increased representation of ATMs in the *Gipr^-/-^* BM epiWAT (**Figure 2B**), and their expression of IL-10 (**Figure 2D**), can explain the upregulation of IL-10 in the *Gipr^-/-^* BM epiWAT SVF (**Figure 1F**).

### Immune Cell-Derived S100A8/A9 Contributes to the Impairment of White Adipose Tissue Type 2 Immune Circuits in *Gipr^−/−^* Bone Marrow Mice

**Figure 3:**
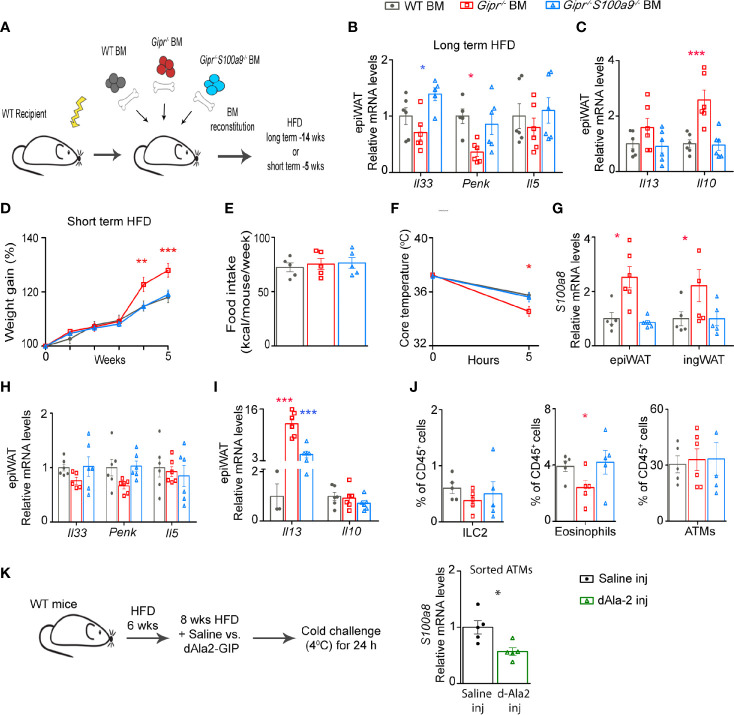
Unrestrained production of S100A8/A9 by *Gipr^-/-^* immune cells impedes WAT type 2 immune circuits. **(A)** Scheme illustrating experimental design. Lethally irradiated WT mice were reconstituted with WT BM (gray circles), *Gipr^-/-^* BM (red squares) and *Gipr^-/-^/S100a9^-/-^* BM (blue triangles) for 6 weeks and then fed with a HFD regime as indicated for **(B**, **C)** long-term (14 weeks) or **(D–J)** short-term (5 weeks). **(B, C)** qRT-PCR assessment of the expression of the indicated mediators normalized to *Rplp0* in epiWAT after 14 weeks of HFD (n=6). **(D, E)** Weekly measurements of body weight and food consumption (kcal/mouse/week) during 5 weeks HFD feeding (n≥6). **(F)** Body core temperature response (°C) to acute cold challenge after 5 h. **(G)** Expression of *S100a8* gene in epiWAT and ingWAT as determined by qRT-PCR. **(H, I)** Gene expression of indicated mediators in epiWAT after 5 weeks of a HFD as analyzed by qRT–PCR and normalized to *Rplp0.* **(J)** Graphs showing flow cytometry assessment of ILC2, eosinophils and ATMs fractions out of total CD45^+^ cell population (defined as shown in **Figure 2A**). **(K)** Scheme illustrating experimental design. WT mice were subjected to HFD feeding for 14 weeks and received daily i.p. injections of dAla2-GIP analogue (60 nmol/kg body weight) or vehicle (saline) during the last eight weeks (n= 8). Left panel, qRT-PCR analysis of *S100a8* expression in ATMs sorted from epiWAT of these mice. Data are from single experiment. Data are presented as mean ± SEM. Statistical analyses were conducted using one-way ANOVA followed by post-hoc Tukey test **(B, C)** or by unpaired two-tailed *t*-test **(K)** with significance: *p ≤ 0.05, **p ≤ 0.01 and ***p ≤ 0.001. Red or blue stars represent significance of *Gipr^-/-^* BM or *Gipr^-/-^/S100a9^-/-^* BM groups vs. WT BM, respectively.

The pro-inflammatory dysregulated metabolic phenotype of *Gipr^-/-^* BM mice has been linked with the unrestrained activity of myeloid cell-derived S100A8/A9 (13). Hence, we next sought to investigate a possible mechanistic link between the alterations in type 2 immunity in the *Gipr^-/-^* BM epiWAT and the hyper-production of S100A8/A9 by GIPR-deficient BM-derived myeloid cells. To test this hypothesis, we studied a group of BM chimera mice reconstituted with BM from *Gipr^−/−^S100a9^−/−^* double knockout mice (*Gipr^−/−^S100a9^−/−^* BM) (**Figure 3A**). *S100a9^−/−^* mice were chosen given the lack of mature S100A8/A9 protein expression in these mice (38). Co-deletion of GIPR and S100A8/A9 reversed the decline in *Il33* and *Penk* expression detected in the epiWAT of *Gipr^-/-^* BM mice at 14 weeks of HFD feeding (**Figure 3B**). S100A8/A9 deletion also restored expression of *Il10* and *Il13* in the epiWAT of *Gipr^-/-^* BM mice to the levels observed in the control WT BM epiWAT (**Figure 3C**).

Time-resolved single cell characterization has recently discovered massive reorganization of the immune cell compartment in mouse epiWAT between 6 and 12 weeks of HFD feeding, with the most prominent changes including an expansion of ATMs and a reduction in type 2 immune cells, including Tregs and ILC2 (39). Hence, we next examined the effect of immune cell-GIPR-deficiency on type 2 immunity at an earlier time point, at 5 weeks of HFD feeding, followed by 5 h cold challenge at 4^0^C to boost thermogenesis activity. Corroborating our earlier findings (**Figure 1A**), *Gipr^-/-^* BM mice exhibited increased body weight starting already at 4 weeks of HFD feeding (**Figure 3D**), despite similar food intake (**Figure 3E**). The co-deletion of S100A8/A9 (*Gipr-/-S100a9-/-*BM) normalized weight gain to that of the control group (**Figure 3D**). We have previously shown that S100A8/A9 directly impairs beiging (13). Indeed, *Gipr-/- *BM chimeric mice displayed reduced core temperature after 5 h of cold challenge, in comparison to the control group, and this defect was ameliorated by the co-deletion of S100A8/A9 (**Figure 3F**), indicating an etiologic role for S100A8/A9 in the failure of these mice to properly adapt thermogenesis. In support of this, the expression of S100A8 (*S100a8*) was profoundly elevated in the epiWAT, and also trended higher in the ingWAT of *Gipr-/-* BM mice, already at this early phase of HFD feeding (**Figure 3G**). Similar to the results obtained during the development of obesity (**Figure 3B**), the reduced expression of *Il33* and *Penk* in the epiWAT of *Gipr-/- *BM mice was restored to WT BM control levels in *Gipr-/-S100a9-/-BM* mice (**Figure 3H**). *Il10* was expressed at similar levels in the epiWAT of WT BM, *Gipr^-/-^* BM and *Gipr-/-S100a9-/-*BM mice after several weeks of HFD feeding. In contrast, *Il13* was profoundly upregulated in *Gipr^-/-^* BM group, and co-deletion of S100A8/A9 partially reverted *Il13* levels towards normal (**Figure 3I**). Flow cytometry analysis revealed a reduction in the fraction of ILC2 and eosinophils in the *Gipr-/-* BM epiWAT, while ATMs were similarly represented at this early phase (**Figure 3J**). We have previously shown that S100A8/A9 originates in the epiWAT SVF cells from ATMs and neutrophils. In line with the role of S100A8/A9 in driving impaired type 2 immunity in the *Gipr-/-* BM epiWAT, we next examined whether treatment with the long-acting GIP analogue (dAla2-GIP) can reduce the S100A8/A9 production in the epiWAT ATMs following 14 weeks of HFD (**Figure 3K**). Indeed, dAla2-GIP administration reduced *S100a8* gene expression in the epiWAT ATMs (**Figure 3K**). Altogether, these results outline a pivotal role for GIP in balancing fat depot type 2 immune networks during the development of obesity, at least partially through its restraining of the immune cell-derived S100A8/A9.

### GIPR-Deficiency in Myeloid Immune Cells Drives the Impairment of Type 2 Immune Networks in the Obese White Adipose Tissue

**Figure 4:**
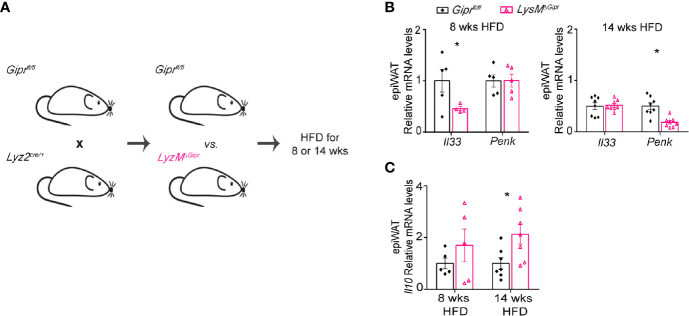
Deficiency of GIP signaling in myeloid immune cells drives the impairment of type 2 immune networks in the obese epiWAT. **(A)** Scheme illustrating experimental design. Mice with targeted GIPR-deficiency to myeloid cells (*LysM* ^ΔGipr^, pink open triangles) and their littermate controls (*Gipr* ^fl/fl^, black rhombus) were fed a HFD for 8 and 14 weeks (n ≥6). **(B)** qRT-PCR assessment of the expression of *Il33* and *Penk* genes in epiWAT normalized to *Rplp0*. **(C)** qRT-PCR assessment of the expression of *Il10* in epiWAT of *LysM* ^ΔGipr^ and *Gipr*fl/fl mice after shorter (8 weeks) and longer (14 weeks) HFD-regimes. Data are analyzed by unpaired, two-tailed *t*-test and presented as mean ± SEM. *p ≤ 0.05.

Myeloid immune cells are the main source of S100A8/A9 during inflammation (40). In the epiWAT from mice with obesity, GIP plays an important role in restraining the production of S100A8/A9 in ATMs (**Figure 3K**) **(** 13), and the deficiency in GIPR in myeloid cells augments myelopoiesis and accumulation of S100A8/A9^+^ neutrophils and ATMs in the epiWAT (13). Therefore, we next examined whether deletion of GIPR specifically in the myeloid cells would be sufficient to induce alteration in type 2 immunity in the epiWAT. Accordingly, conditional *Gipr^fl/fl^* mice were crossed with *Lyz2^cre/+^* mice. The resulted *LysM^ΔGipr^* and their littermate *Gipr^fl/fl^* controls were subjected to eight and 14 weeks HFD regimens (**Figure 4A**). Similarly to *Gipr-/-* BM, *LysM^ΔGipr^* mice exhibit an aggravated metabolic phenotype in response to prolonged HFD, associated with increased weight gain, steatohepatitis, insulin resistance, myelopoiesis and S100A8/A9 production, as well as impaired ingWAT beiging (13). Compared to their *Gipr^fl/fl^* littermate controls, *LysM^ΔGipr^* mice displayed reduced levels of *Il33* in the epiWAT at 8 weeks and of *Penk* at 14 weeks of HFD feeding (**Figure 4B**). Similar to the results in *Gipr-/-* BM mice, increased expression of *Il10* was detected in the epiWAT of *LysM^ΔGipr^* mice compared to *Gipr^fl/fl^* littermate controls, but only after 14 weeks of HFD feeding (**Figure 4C**). Altogether, these results further consolidate an immunoregulatory role for GIP in myeloid cells.

### GIPR-Deficiency in Immune Cells Alters the Expression of Type 2 Immune Mediators by Cells in the Adipocyte Fraction

**Figure 5:**
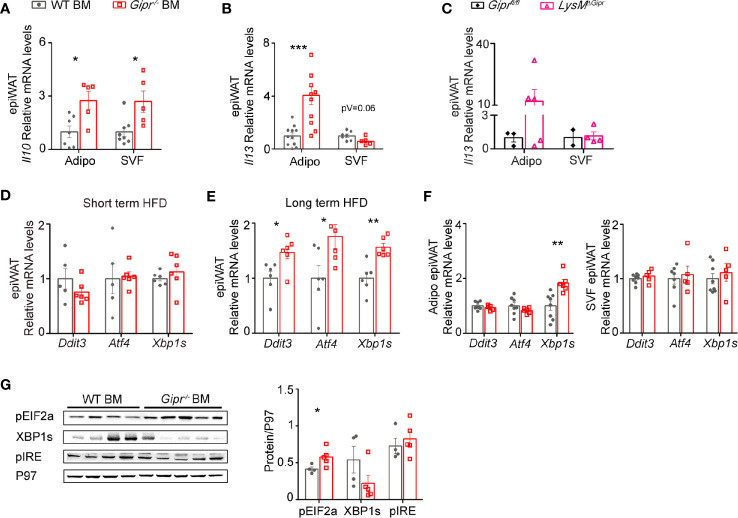
Impairment of type 2 immune networks in *Gipr^-/-^* BM epiWAT involves adipocyte fraction cells. Expression of *Il10* **(A)** and *Il13* **(B)** in isolated adipocyte fraction and SVF in epiWAT of WT BM (gray circles) or *Gipr^-/-^* BM (red squares) mice after 14 weeks of HFD as determined by qRT-PCR analysis. **(C)** qRT-PCR assessment of the expression of *Il13* in isolated adipocyte fraction and SVF in epiWAT of *LysM* ^ΔGipr^ (pink open triangles) and *Gipr* ^fl/fl^ (black rhombus) obese mice after 14 weeks of HFD. **(D**, **E)** qRT-PCR assessment of the expression of indicated genes in epiWAT at shorter (5 weeks) and longer (14 weeks) HFD-regimes respectively, normalized to *Rplp0*. **(F)** qRT-PCR assessment of the expression of indicated genes in isolated adipocyte fraction and SVF in epiWAT of WT BM or *Gipr^-/-^* BM mice after 14 weeks of HFD. **(G)** Expression of phosphorylated EIF2 (pEIF2α), spliced form of XBP1 (XBP1s) and phosphorylated IRE (pIRE) in epiWAT from WT BM (n=4) and *Gipr^-/-^* BM (n=5) mice, as assessed by immunoblot analysis and was quantified using P97 as loading control (for pEIF2α, P=0.04, for XBP1s, p=0.15 and for pIRE, P=0.54), with densitometry presented below. Data are analyzed by unpaired, two-tailed *t*-test, and results are presented as mean ± SEM with significance: *p < 0.05, **p < 0.01, ***p < 0.001. Data in panel **(A)** are from two independent experiments, and in **(B–F)** are from one experiment. Adipo, adipocyte fraction; SVF, stromal vascular fraction.

In addition to WAT immune cells, adipocytes can also be a source for type 2 immune cytokines, such as IL-13 (41–43). Hence, we next examined the expression of *Il13* in the WAT of WT BM vs. *Gipr^-/-^* BM after 14 weeks of HFD feeding, while distinguishing between the adipocyte fraction and the SVF. Levels of mRNA transcripts for the beiging-interfering cytokine *Il10* were profoundly elevated in both SVF and adipocyte fractions of the *Gipr^-/-^* BM WAT (**Figure 5A**). Notably, *Il13* expression was reduced in the SVF (p=0.06), but highly increased in the adipocyte fraction of the *Gipr^-/-^* BM WAT (**Figure 5B**), a phenomena associated with increased WAT inflammation during obesity (42). Moreover, we detected increased expression of *Il13* in the adipocyte fraction, but not the SVF, of the *LysM^ΔGipr^* epiWAT (**Figure 5C**).

Previous studies have shown that expression of IL-10 and IL-13 in WAT cells may be elevated by obesity-induced ER stress (43, 44). There are three major ER stress sensors, IRE1, PERK and ATF6, which transduce the mammalian unfolded protein response (UPR). Activated IRE1 splices the mRNA of XBP1 yielding the potent transcription factor spliced XBP1 (XBP1s). Activated PERK phosphorylates EIF2α (pEIF2α), which induces translation of ATF4 that further acts as a transcription factor for *Ddit3* (45). ER stress/UPR- related gene expression was assessed in the total epiWAT of WT BM and *Gipr^-/-^* BM mice after both short (5 weeks) and long-term (14 weeks) HFD regimens. Expression of ER stress/UPR markers was not altered after short-term HFD feeding in the epiWAT of *Gipr^-/-^* BM mice compared to expression in control WT BM mice (**Figure 5D**). In contrast, after 14 weeks of exposure to HFD, the expression of *Atf4*, *Ddit3*, and *Xbp1s*, were all enhanced in the epiWAT of *Gipr^-/-^* BM mice (**Figure 5E**). Fractionation of total epiWAT revealed that mRNA expression of *Xbp1s* was elevated in the adipocyte fraction of *Gipr^-/-^* BM mice, but not in the SVF (**Figure 5F**). Western blot analysis revealed an increased phosphorylation of pEIF2α in the epiWAT of *Gipr^-/-^* BM mice (**Figure 5G**). In contrast, despite the increased gene expression of *Xbp1s*, the protein levels of XBP1s were reduced, and the expression level of its upstream activator pIRE did not vary (**Figure 5G**). pEIF2α is also required for expression of maximal stable spliced XBP1 protein *via* a mechanism that involves stabilization of *Xbp1s* mRNA (46). Hence, the increased expression of pEIF2α may explain the increase in *Xbp1s*. Altogether, these results reveal that absence of the GIPR signaling in immune cells alters the expression of type 2 immune mediators in adipocytes, associated with the induction of the PERK-EIF2a ER stress pathway during the development of obesity.

### A Glucose-Dependent Insulinotropic Polypeptide Analogue Induces IL-33 and Penk in Epididymal Visceral White Adipose Tissue of Lean Mice During Cold-Induced Thermogenesis

**Figure 6:**
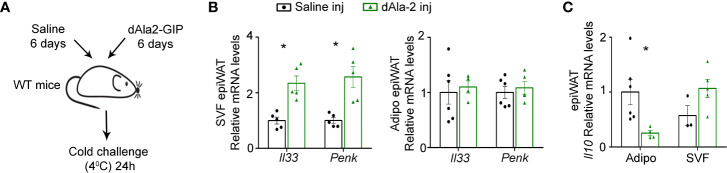
GIP analogue enhances expression of type 2 mediators in epiWAT of lean mice during cold-induced thermogenesis. **(A)** Scheme illustrating experimental design. WT mice (n= 6) received daily i.p. injections of dAla2-GIP analogue (60 nmol/kg body weight) or vehicle (saline) for 6 days and after that were subjected to cold challenge (4°C) for 24 h. **(B)** qRT-PCR assessment of the expression of *Il33* and *Penk* genes in isolated adipocyte fraction and SVF of epiWAT normalized to *Rplp0*. **(C)** Expression of *Il10* in isolated adipocyte fraction and SVF of epiWAT of these mice was analyzed by qRT-PCR and normalized to *Rplp0*. Data are from a single experiment and analyzed by unpaired, 2-tailed *t*-test and presented as mean ± SEM. *p ≤ 0.05.

To further substantiate the association between GIP and WAT type 2 immunity we next studied the impact of treatment with a GIP analogue on the production of type 2 immune mediators. IL-33 and Penk, both key stimulators of adaptive thermogenesis (23), were reduced in *Gipr^-/-^* BM epiWAT at both short (5 weeks) (**Figure 3H**) and long term (14 weeks) (**Figure 1D**) HFD. Hence, we next assessed the effect of GIP analogue treatment on IL-33 and Penk production in lean mice subjected to cold challenge (4°C). Accordingly, 7 weeks old C57BL/6J OlaHsd mice received daily i.p. injections of dAla2-GIP or vehicle (saline) for 6 days, and then were placed at 4°C for 24 h (**Figure 6A**). GIP treatment was sufficient to promote the expression of both *Il33* and *Penk* in the SVF, but not in the adipocyte fraction (**Figures 6B, C** **)**. Given the increased expression of the beiging-interfering cytokine IL-10 in the epiWAT of *Gipr^-/-^* BM (**Figure 1F**) and *LysM^ΔGipr^* mice (**Figure 4C**) under prolonged HFD, we next examined the effect of the GIP analogue on cytokine expression in response to cold-challenge. Interestingly, dAla2-GIP treatment reduced IL-10 expression uniquely in the epiWAT adipocyte fraction, but not in the SVF fraction (**Figure 6C**). These results further substantiate a role for GIP in modulation of type 2 immune circuits in the epiWAT.

## Discussion

Here we uncover a new gut-immune axis showing that an absence of GIP signaling in immune cells during HFD hampers the production of key type 2 immune mediators and leads to the reduction of type 2 immune cells in the epiWAT, concomitantly with the augmented production of the beiging interfering cytokine IL-10 in both epiWAT adipocyte and SVF fractions. This impairment initiates already at the early stage of HFD feeding.

The WAT cellular ecosystem harbors, in addition to resident adipocytes and their mesenchymal progenitors, a unique blend of accessory immune cells, all intimately cooperating to regulate metabolic homeostasis (1, 17). During obesity, acute self-limiting inflammation allows healthy expansion of adipose tissue and adaptation to the altered metabolic demands derived from over-nutrition. However, in the course of a prolonged positive energy surplus, this physiological response becomes sustained and turns pathogenic, leading to pro-inflammatory transition of WAT resident and infiltrating immune cells (47). Type 2 immune cell networks are an essential factor directly affecting metabolic homeostasis and energy balance in WAT and limiting WAT inflammation during obesity. The numbers of WAT type 2 immune cells, such as ILC2, eosinophils, and Tregs, decline with progressing adiposity, thereby licensing obesity-induced inflammatory transition in the WAT (22, 39). We show here that the increased adiposity in *Gipr^-/-^* BM mice is associated with the impaired production of key type 2 immune mediators that are directly involved with both metabolic and immunoregulatory activities that balance nutrient overload. In this respect, *Gipr^-/-^* BM mice exhibit reduced expression of mediators that are indispensable for proper beiging response, such as IL-33 and Penk (24, 26). IL-33 is also crucial for the maintenance of WAT ILC2 and Tregs expressing its receptor (21, 23–25), and indeed there was a significant decline in the fraction of these cells as well as in eosinophils in the epiWAT of *Gipr^-/-^* BM mice. Importantly, we show that the alteration in type 2 immune networks in *Gipr^-/-^* BM mice appears early during the development of obesity, subsequent to the acquisition of increased weight gain. Therefore, we believe that this impairment in type 2 immune pathways fuels the imbalanced energy expenditure and dysregulated beiging process previously linked to the GIP signaling loss in immune cells during DIO (13). Of note, we have not examined whether the alteration in type 2 immunity occurs prior to the increased weight gain, and therefore it remains unclear whether it is the initiating event disrupting energy expenditure. Moreover, the increased weight gain and associated inflammation may contribute to the impairment of type 2 immune networks in *Gipr^-/-^* BM mice. Importantly, we show here that alteration in type 2 immune networks in *Gipr^-/-^* BM mice appear mainly in the epiWAT, but not ingWAT. Hence, it remains mechanistically unclear whether and how these alterations in the epiWAT are associated with the impaired ingWAT beiging response previously reported in these mice (13).

Our previous studies have outlined an etiologic role for the inflammatory alarmin S100A8/A9 in the aggravated metabolic phenotype observed in *Gipr^-/-^* BM mice. Accordingly, the co-deletion of S100A8/A9 in *Gipr^-/-^* immune cells ameliorated the increased weight gain and impaired beiging displayed by *Gipr^-/-^* BM chimeric mice during HFD feeding (13). Moreover, a single S100A8/A9 injection was sufficient to impair the beiging response in cold-challenged lean WT mice (13). Here we provide an extended mechanistic insight into the importance of GIP-governed restraint of S100A8/A9 on the maintenance of energy balance, showing that S100A8/A9 drives some of the alterations in the production of type 2 immune mediators in the epiWAT of HFD-fed *Gipr^-/-^* BM mice. We show that *S100a8* gene expression is already increased in both epiWAT and ingWAT after 5 weeks in HFD-fed mice, and that co-deletion of S100A8/A9 restores the expression of type 2 mediators and the representation of type 2 immune cells to that of the control WT BM chimera mice. This coupling between S100A8/A9 overproduction by myeloid cells in the context of disrupted GIP signaling and the impairment of whole-body energy homeostasis and type 2 immunity has immediate translational relevance, as elevated S100A8/A9 expression in VAT and serum is also detected in people with type 2 diabetes mellitus (T2DM) and obesity (48, 49). Therefore, it will be necessary to determine further how S100A8/A9 affects type 2 immunity in the WAT. This may be challenging given that the S100A8 and S100A9 calgranulins can also appear as homodimers, which may differentially regulate adaptive thermogenesis either *via* effects on specific immune cells and/or *via* direct effects on beiging differentiation pathways in adipocytes. Moreover, S100A8, S100A9, and S100A8/A9 calgranulins can signal *via* multiple receptors, such as TLR4, RAGE, and Basigin (CD147) (50), which may be expressed by a broad spectrum of cells in the WAT.

Emerging studies outline direct immunoregulatory properties for GIP that are intertwined with its metabolic actions (6, 7). Therefore, there is a tremendous unmet need to dissect the exact repertoire of immune cells capable of sensing GIP, their location, and the resulting impact on central and peripheral metabolism. Mice with conditional targeting of GIPR-deficiency to myeloid immune cells exhibit abnormal metabolic phenotypes in response to sustained HFD feeding similar to that of *Gipr^-/-^* BM mice (13), suggesting that the GIP signaling is especially essential in myeloid cells for maintaining energy balance and tuning WAT inflammation. We have previously shown and corroborated here, that both *in vivo* and directly *ex vivo* treatments with a GIP analogue attenuate the expression of *S100a8* gene in ATMs from the obese epiWAT. In alignment with this, a direct anti-inflammatory role for GIP in macrophages has also been suggested in the context of atherosclerosis (51–53), as well as in microglia in neurodegenerative disorders of the central nervous system (54–58). It remains unclear however, whether WAT immune cells, specifically type 2 immune cells, directly respond to GIP. The lack of a reliable GIPR antibody has been a barrier in mapping cell-specific GIPR protein expression. Hence, GIPR expression is often determined at the transcript level combined with functional validation for GIP-induced Ca^2+^ influx and cAMP levels. Its expression in low copy number further excludes it from single cell Atlases of WAT immune and non-immune cells. Relative to the abundance of WAT adipocytes and ATMs, the scarcity of type 2 immune cells limits functional screenings of *Gipr* expression, therefore necessitating the generation of reporter mouse models and modified ligands that permit receptor labelling. Recently, transgenic mice allowing the conditional *Gipr* gene deletion in broad immune cell populations were used to map *Gipr* mRNA transcripts in various tissues. The immune expression of *Gipr* mRNA transcripts was most prominent in the BM, mainly among T cells, Gr1^+^CD11b^+^ myeloid cells and myeloid progenitor cells (7). Notably, the transgenic deletion of the *Gipr* gene in immune cells had no clear effect on the expression of *Gipr* mRNA transcripts in the lean epiWAT and mesenteric WAT, therefore challenging the idea that WAT type 2 immune cells are directly responding to GIP and suggesting the BM as the site for GIP immunoregulatory activity. Hence, the metabolic phenotypes observed within the WAT of HFD-fed *Gipr^-/-^* BM mice and *LysM^ΔGipr^* mice may be mediated by the lack of GIPR signaling in myeloid immune cells in the BM. In this respect, both Gipr*^-/-^* BM mice and *LysM^ΔGipr^* mice exhibit evidence for myelopoiesis and especially neutrophilia in peripheral blood and epiWAT during HFD, which may explain the increased expression of S100A8/A9 and its governed deleterious metabolic and inflammatory effects.

Utilizing a gain of function approach, we show here that treatment with the long-acting GIP analogue dAla2-GIP in weight-matched lean mice prior to their exposure to cold-induced thermogenesis resulted in increased expression of type 2 immune mediators IL-33 and Penk, both pivotal drivers of adaptive thermogenesis (23). These mediators are mainly produced by mesenchymal stromal cell subtypes (27, 28), suggesting non-immune pathway by which GIP can modulate type 2 immunity. Nevertheless, it remains elusive whether these cells can indeed directly recognize and respond to GIP. While loss-of-function studies indicate that GIP drives weight gain, when it comes to GIPR agonism and antagonism, compelling evidence paradoxically indicate that both approaches can reduce body weight, especially if combined with GLP-1 agonists (12, 59). This paradox may reflect the partial agonistic activity of certain antagonists (60), compensatory relationship and overlapping signaling axes between GIPR and GLP-1R incretin receptors (59, 61), as well as desensitization of GIPR achieved by chronic agonism (62). With respect to the latter, in cultured mouse or human preadipocytes, exposure to 1 mM dAla2-GIP for 24 h decreased cAMP response to subsequent GIPR stimulation up to 48 h (62), indicating that chronic GIPR agonism functionally mimics GIPR antagonism. Therefore, it will be important to reconcile whether the GIP augmentation approach used here to achieve upregulation of IL-33 and Penk arises *via* GIPR agonism or antagonism. Noteworthy, despite the emerging concept that systemic GIP antagonism has a positive effect on body weight, similarly to the whole-body loss-of-function phenotypes of GIPR or GIP-deficiencies, targeting of GIPR-deficiency to myeloid immune cells produces the opposite effect with impaired energy expenditure and increased weight gain during nutrient overload. Hence, understanding the mechanisms behind the positive effects on body weight of therapies based on GIP analogues may benefit from incorporating knowledge of how GIPR signaling impacts different cell types within the immunological compartment, and specifically type 2 immunity.

In conclusion, we identified GIPR signaling as an important regulator of type 2 immunity networks in epiWAT and energy expenditure in ingWAT and a gatekeeper of the unrestrained activity of S100A8/A9 in myeloid cells during obesity. Our findings outline a pivotal gut-immune axis mediated by GIP in controlling immunometabolic crosstalk in response to energy intake.

## Data Availability Statement

The original contributions presented in the study are included in the article. Further inquiries can be directed to the corresponding authors.

## Ethics Statement

The animal study was reviewed and approved by The Animal Care Use Committee of the Sourasky Medical Center.

## Author Contributions

The corresponding authors (CV and SF) confirm that all authors agreed to be accountable for the content of the work. IE, IZ, SF, and CV conceived the study, designed the experiments, and wrote the manuscript. IE, IS, IZ, and DM performed the experiments and analyzed the data. AN performed some of the RT–PCR and immunoblot analyses in the presented experiments. DD provided key scientific consultation, mouse tools, and carefully reviewed and edited the manuscript. CV and SF supervised the study. All authors contributed to the article and approved the submitted version.

## Funding

CV and SF have received funding from the Israeli Science Foundation (grant nos. 1146/16 and 1114/20). CV is supported by the Joint Canada-Israel Health Research Program (grant no. 3416/19). DD is supported by a Banting and Best Diabetes Centre-Novo Nordisk Chair in Incretin Biology, the Novo Nordisk Foundation-Sinai Health Fund in Regulatory Peptides, CIHR grant 154321. These studies were directly supported by the Canada-Israel Health Research Initiative, jointly funded by the Canadian Institutes of Health Research, the Israel Science Foundation, International Development Research Centre, and the Azrieli Foundation, project 109150. Funding sources were not involved in the study design, collection, analysis, interpretation of data, preparation of the manuscript, and its submission.

## Conflict of Interest

DD has served as an advisor or consultant or speaker within the past 12 months to Boehringer Ingelheim Inc., Forkhead Biotherapeutics, Intarcia Therapeutics, Kallyope, Merck Research Laboratories, Eli Lilly Inc., Novo Nordisk Inc. Neither DD nor his family members hold stock directly or indirectly in any of these companies. GLP-2 is the subject of a patent license agreement between Shire Inc and the University of Toronto, Toronto General Hospital (UHN), and Daniel Drucker.

The remaining authors declare that the research was conducted in the absence of any commercial or financial relationships that could be construed as a potential conflict of interest.
